# Seasonally-reversed trends in the subtropical Northwestern Pacific linked to asymmetric AMO influences

**DOI:** 10.1038/s41598-023-40979-9

**Published:** 2023-08-23

**Authors:** Yong-Fu Lin, Chuen-Teyr Terng, Chau-Ron Wu, Jin-Yi Yu

**Affiliations:** 1 Department of Earth System Science, University of California, Irvine, CA USA; 2 Central Weather Bureau, Taipei, Taiwan; 3 Department of Earth Sciences, National Taiwan Normal University, Taipei, Taiwan; 4 Research Center for Environmental Changes, Academia Sinica, Taipei, Taiwan

**Keywords:** Climate sciences, Ocean sciences

## Abstract

This study identifies seasonally-reversed trends in Kuroshio strength and sea surface temperatures (SSTs) within the western North Pacific (WNP) since the 1990s, specifically in the 22° N–28° N region. These trends are characterized by increases during summer and decreases during winter. The seasonally-reversed trends are a result of the asymmetric responses of the WNP to a shift towards the positive phase of the Atlantic multidecadal oscillation (AMO) around the same period. The positive AMO induces an anomalous descent over the North Pacific during summer, leading to the direct strengthening of the gyre. However, during winter, it triggers an anomalous descent over the tropical Pacific, which excites a poleward wavetrain impacting the WNP and causing gyre weakening. The associated responses of the East Asian monsoon and China Coastal Current contribute to the observed seasonally-reversed SST trends. It is noteworthy that the seasonally-reversed trends in gyre strength and SSTs are predominantly observed north of 20° N in the WNP. This limitation arises because the anomalous cyclone within the winter poleward wavetrain is located north of this latitude boundary. Specifically, the clearest trends in gyre strength are observed in the northern segment of the Kuroshio, while the manifestation of SST trends in the Taiwan Strait could potentially be attributed to the influence and enhancement of the East Asian monsoon and the China Coastal Current. Due to the limited length of observational data, statistical significance of some of the signals discussed is rather limited. A CESM1 pacemaker experiments is further conducted to confirm the asymmetric responses of the North Pacific to the AMO between the summer and winter seasons.

## Introduction

An increasing number of studies indicate that Pacific climate has experienced significant changes since the 1990s^1–4^. In the tropical Pacific, the location of El Niño-Southern Oscillation (ENSO) events moved from the eastern Pacific to the central Pacific^4–9^. Beginning around the same time, there was a southward migration of the mean locations of the North Equatorial Current (NEC) and the North Equatorial Counter Current (NECC)^2^. Sea level rise in the western Pacific have also accelerated during this period^1^. Furthermore, the interannual variability in monsoon rainfall has significantly decreased since 1993^3^.

In the western North Pacific (WNP), the North Pacific subtropical gyre (NPSG) is a key feature that shapes regional climate. Variations in the strength of the NPSG modulate regional circulations in the East China Sea and South China Sea, the strengths of the Mindanao, Taiwan Strait, and Luzon Strait currents^10–12^, typhoon development^13^, and even fish migration^14–20^. For example, a weakening (strengthening) of the Kuroshio, which is the western boundary current of the NPSG, can induce more (less) intrusion events into East China Sea and South China Sea, affecting fish migration, as well as mass, heat, salinity, and nutrient balances^11,12,21–25^. As another example, a weakening (strengthening) of the NEC component of the NPSG can weaken (strengthen) the Mindanao Current^26^.

**Figure 1:**
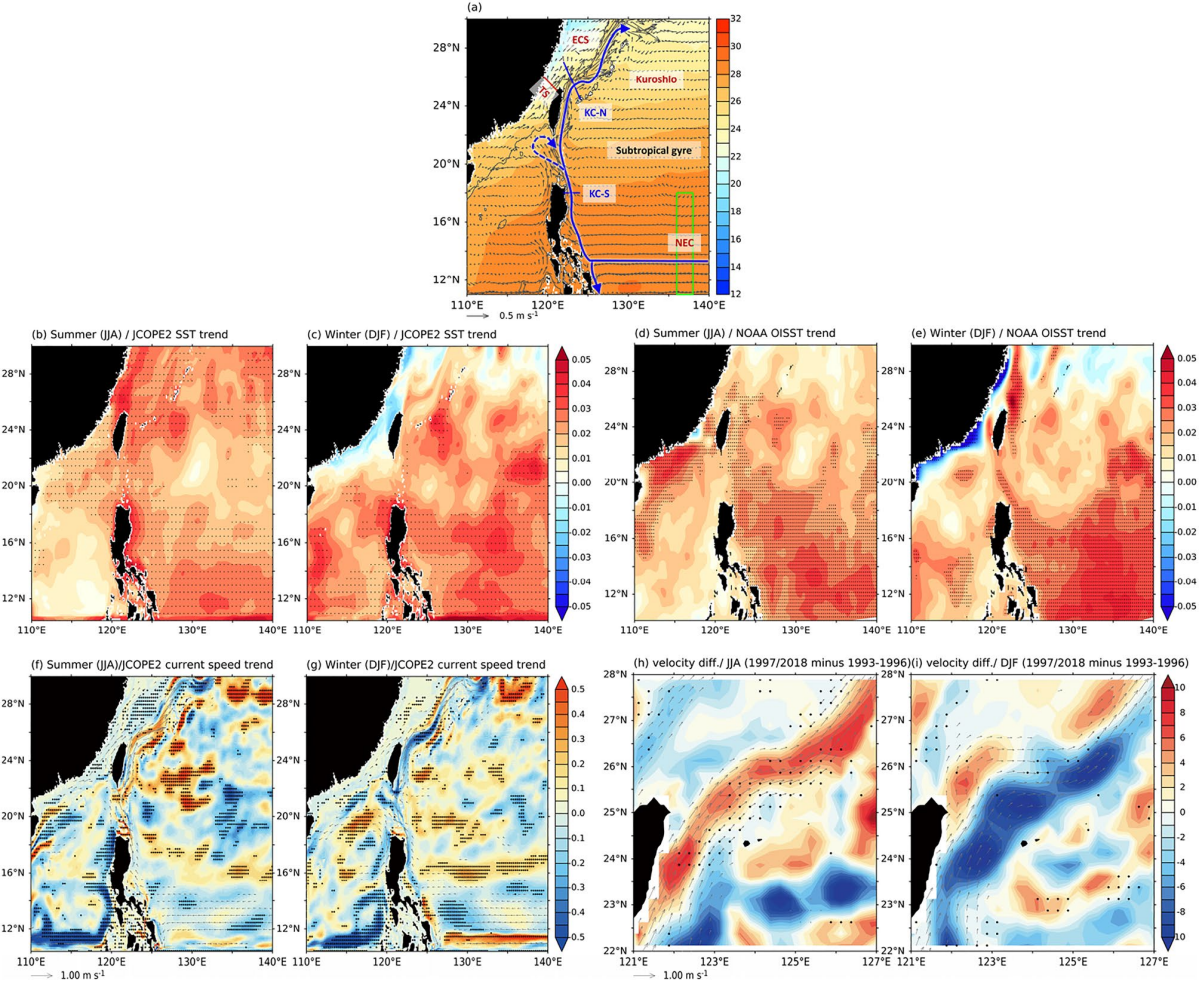
(**a**) Climatology of sea surface temperature (colors, in °C) and upper-ocean (0–200 m) current speed (vectors) from the JCOPE2 reanalysis data during the period 1993–2018; (**b,c**) boreal summer and winter linear trends in SSTs (in °C/year) of the JCOPE2 reanalysis data and (**d,e**) the NOAA OISST observational data. (**f,g**) boreal summer and winter linear trends in currents of the JCOPE2 reanalysis data during the period; and (**h,i**) summer and winter geostrophic surface velocity differences (color, in cm/s) between two sub-periods (1997–2018 minus 1993–1996) of the AVIOS observational data. In (**a**), the bold blue arrow marks the mean location of the North Equatorial Current (NEC)-Kuroshio component of the North Pacific Subtropical Gyre. Also marked are the locations of the Taiwan Strait (TS), East China Sea (ECS), South China Sea (SCS), Mindanao Current (MC), and the south and north segments of the Kuroshio (KC-S and KC-N, respectively). The blue line next to KC-N marks the area (24.5–27° N and 122–124° E) used to calculate the area-average KC-N current speed. Vectors are the summer and winter current climatology calculated from the JCOPE2 reanalysis for the period 1993 to 2018. In (**f–i**), the vectors represent the summer and winter current climatology. Only currents exceeding 10 cm/s in (**f,g**) and 20 cm/s in (**h,i**) are shown. These climatologies were calculated from the JCOPE2 and AVISO data for the period 1993 to 2018. In (**b–i**), stippled areas indicate significance at the 90% confidence level determined using a Student’s t-test.

In this study, we focus on contrasting the summer and winter trends in the WNP since the 1990s with a particular focus on the region around the Taiwan Strait (Fig. 1a). The strait is a narrow passage connecting the broad and shallow East China Sea to the north of the Strait to the much deeper South China Sea to the south. It is located right off the East Asian continent, and the currents and sea surface temperatures (SSTs) there can be influenced by trends in the East Asian monsoon^27^. Over the region, southwestly monsoon winds dominate during the summer and northeastly monsoon winds dominate during the winter.

In addition to the Pacific Decadal Oscillation (PDO), the WNP can be influenced by the Atlantic Multi-decadal Oscillation (AMO) over decadal or longer time scales via trans-basin processes. Previous studies have suggested that the AMO can influence the North Pacific climate either directly through the mid-latitudes^28–30^ or indirectly through the tropics^30–34^. In this study, we show with observational analyses and pacemaker coupled model experiments that trends (since the 1990s) in SSTs and the NPSG strength in the WNP have an interesting seasonally-reversed character due to different trans-basin influences from the Atlantic Ocean between summer and winter.

## Results

### Trends in the western North Pacific SSTs and currents during 1993–2018

We first examined the summer (June–July–August; JJA) and winter (December–January–February; DJF) linear trends in SST and upper-ocean (0–200 m) current speed in the WNP during 1993–2018. While warming trends are found during both summer and winter in most of the WNP, a summer warming trend but a winter cooling trend appear off the East Asian Coast to the north of 20° N (Fig. 1b,c). This phenomenon of seasonally-reversed trends around the Taiwan Strait is verified by the high-resolution OISST data (Fig. 1d,e). A similar seasonal reversal is also found in the trends in upper-ocean current speeds along the Kuroshio component of the NPSG (Fig. 1f,g), which exhibit a strengthening trend during summer but a weakening trend during winter, with the most noticeable effect occurring in the 22° N–28° N region.

To further examine the current trend, we divided the Kuroshio into two sectors (see Fig. 1a): the southern sector (KC-S; hereafter) that is upstream of the Taiwan Strait and the northern sector (KC-N; hereafter) that is downstream of the Taiwan Strait. As depicted in Fig. 1f,g, the seasonally reversed trends most apparent in the KC-N sector, still observable south of KC-N along the Kuroshio from 22° to 28° N, diminishing south of Taiwan, and fading over the KC-S sector. Smaller weakening trends are found in KC-S and NEC during both summer and winter. Most pronounced seasonally-reversed trends from 22° to 28° N in the WNP, including the Taiwan Strait and KC-N sector of the Kuroshio.

We further quantify in Fig. S1 the seasonal trends in the Taiwan Strait SST and two major currents of the North Pacific subtropical gyre near the Taiwan Strait (i.e., the NEC and Kuroshio) (see Fig. 1a). We examine the current trends in KC-S using the current averaged around 18° N and 122–124° E and the current trends in KC-N using the current averaged around 24.5–27° N and 122–124° E. We find weak linear trends in the NEC for both the summer (0.0008 cm/s/year, P > 0.1) and winter (− 0.02 cm/s/year, P > 0.1) seasons (Fig. S1a,b). The seasonally-reversed trends that we observed around the Taiwan Strait did not occur in the NEC. The current trends of KC-S (Fig. S1c,d) are similar to the NEC trends, which are very weak during summer (0.005 cm/s/year, P > 0.1) and slightly negative during winter (trend =  − 0.06 cm/s/year, P > 0.1). However, a seasonally-reversed trend appears in the KC-N segment of the Kuroshio (Fig. S1e,f). An increasing current trend is found in the summer (0.07 cm/s/year, P > 0.1), but there is a decreasing trend in the winter (− 0.05 cm/s/year, P > 0.1). Although the trends are not statistically significant, they provide indications that KC-N segment of the Kuroshio have seasonally reversal trends. This phenomenon also occurs in the SST of the Taiwan Strait. The variations in the SST anomalies, after being averaged across the TS transect line, indicate a linear warming trend of 0.29 °C/decade (P < 0.01) during the summer (Fig. S1g) and a cooling trend of − 0.17 °C/decade (P > 0.1) during the winter from 1993 to 2018 (Fig. S1h). The cooling trend is not statistically significant because it is affected by strong interannual variability, such as ENSO. Nonetheless, it shows that the Taiwan Strait has become cooler, exhibiting the opposite trend compared to summer.

Our analyses indicate that the seasonally-reversed trend appears in the Taiwan Strait SSTs and Kuroshio strength. A large-scale driving mechanism is likely to exist to cause such a seasonally-reversed trend. Our analysis of the different segments of the Kuroshio also suggests that the reversed trend does not exist in the equatorial part of the NWP but becomes apparent somewhere around 22° N–28° N. The driving mechanism has to explain not only the seasonally-revering feature of the trend but also this latitudinal dependence of the forcing.

### Trans-basin influences of the Atlantic Ocean on the Pacific climate

To uncover the large-scale driving mechanism behind the reversed trends described in “Trends in the western North Pacific SSTs and currents during 1993–2018” section, we first calculated the correlation coefficients between monthly upper-ocean (0–200 m) Kuroshio Current speed averaged along the KC-N line (see Fig. 1a) and wind stress curl (WSC) over the Western North Pacific during 1993–2018. The correlation (Fig. S2) indicates that WSC anomalies (WSCAs) over 125° E–165° W and 18–35° N of the WNP are most correlated to surface current variations in the KC-N sector.

**Figure 2:**
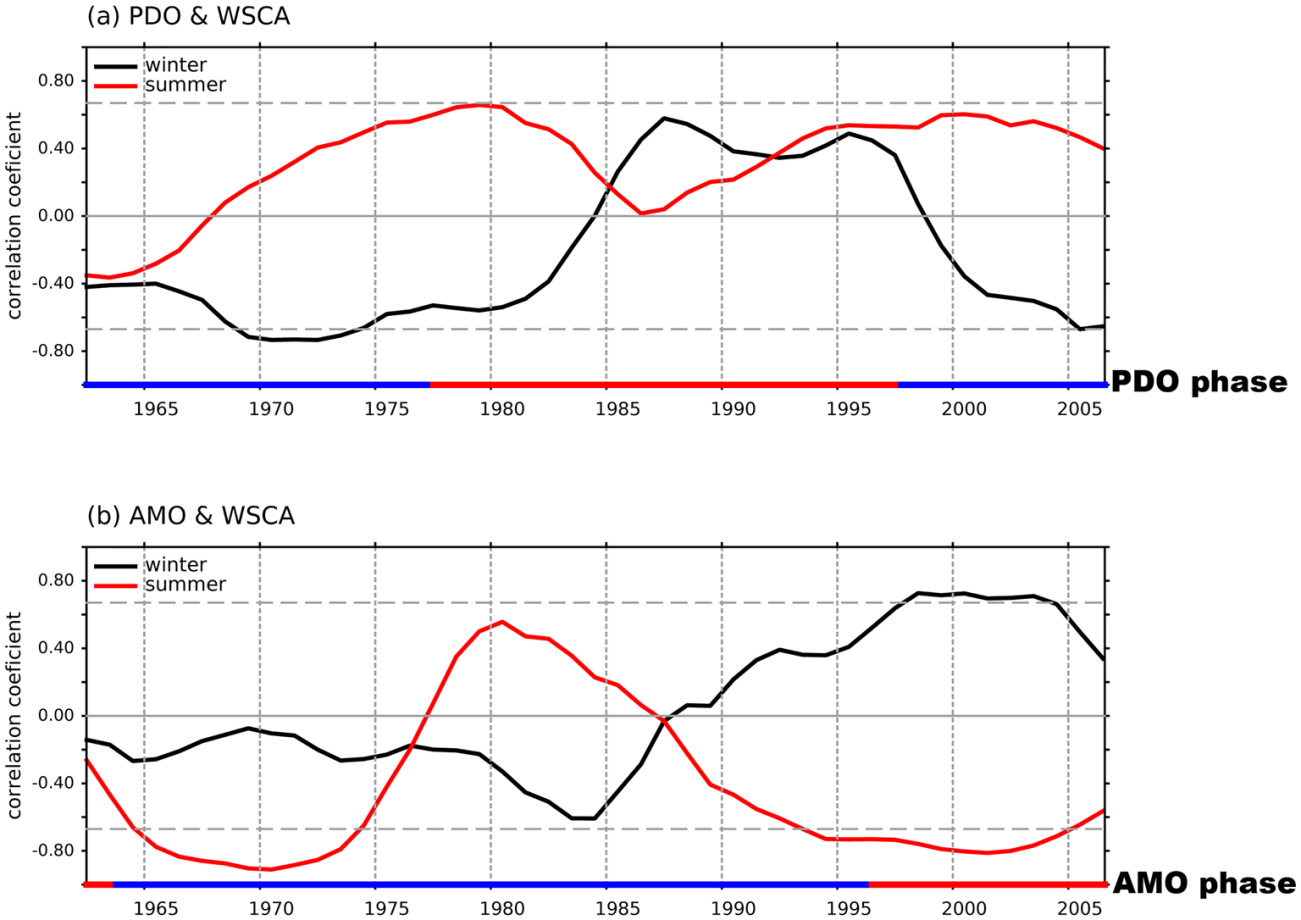
The 21-year running correlation between the yearly (**a**) Pacific decadal oscillation (PDO) and (**b**) Atlantic multi-decadal oscillation (AMO) index with the wind stress curl anomalies averaged over the region of the Western North Pacific (125° E–165° W an 18–35° N, shown by the green box in Fig. S3) during summer (red line) and winter (black line). Grey-dashed lines mark the 90% confidence level determined using a Student’s t-test. The red and blue shadings at bottom of (**a,b**) respectively indicate the positive and negative phases of the PDO and AMO determined using the 7-year running mean values of the indices.

We then calculated the 21-year running correlations between the summer and winter WSCA averaged over this region and the PDO and AMO indices during 1948–2018 to explore the roles of these two decadal variability modes in the current trends. We found that the summer and winter WSCA correlations with the PDO index never pass the 90% significance interval during the analysis period (Fig. 2a), while the correlations with the AMO index both exceeds this interval after the 1990s (Fig. 2b). Most interestingly, the summer WSCA and winter WSCA show reversed correlations with the AMO index during the decades after the 1990s. The AMO has a significant negative correlation with the summer WSCA after the 1990s but a significant positive correlation with the winter WSCA. The 1990s is the time when the AMO switched from a negative phase to a positive phase (Fig. S3). Based on the correlations, the positive AMO phase since the 1990s is expected to result in negative summer WSCAs over the WNP, which strengthen the gyre circulation within the region where the WSCAs were averaged. Similarly, during the same positive AMO phase, positive winter WSCAs are anticipated to weaken the gyre circulation in the region. The seasonally reversed trends in the WNP region since the 1990s can be explained as a result of the seasonally-asymmetric responses of the WNP wind stress curl to the positive phase of the AMO.

To further verify that the AMO can induce the seasonally reversed trends in the Kuroshio, we used the geostrophic surface current velocity derived from AVISO observational data to examine the velocity differences between a negative-AMO period (i.e., 1993–1996) and a positive-AMO period (i.e., 1997–2018) during both summer and winter (Fig. 1h,i). The figures also show the summer and winter current climatologies, averaged from 1993 to 2018. The differences observed confirm that surface currents in the Kuroshio, have strengthened during summer (Fig. 1h) and weakened during winter (Fig. 1i) after the AMO changed from a negative to positive phase around the mid-1990s.

**Figure 3:**
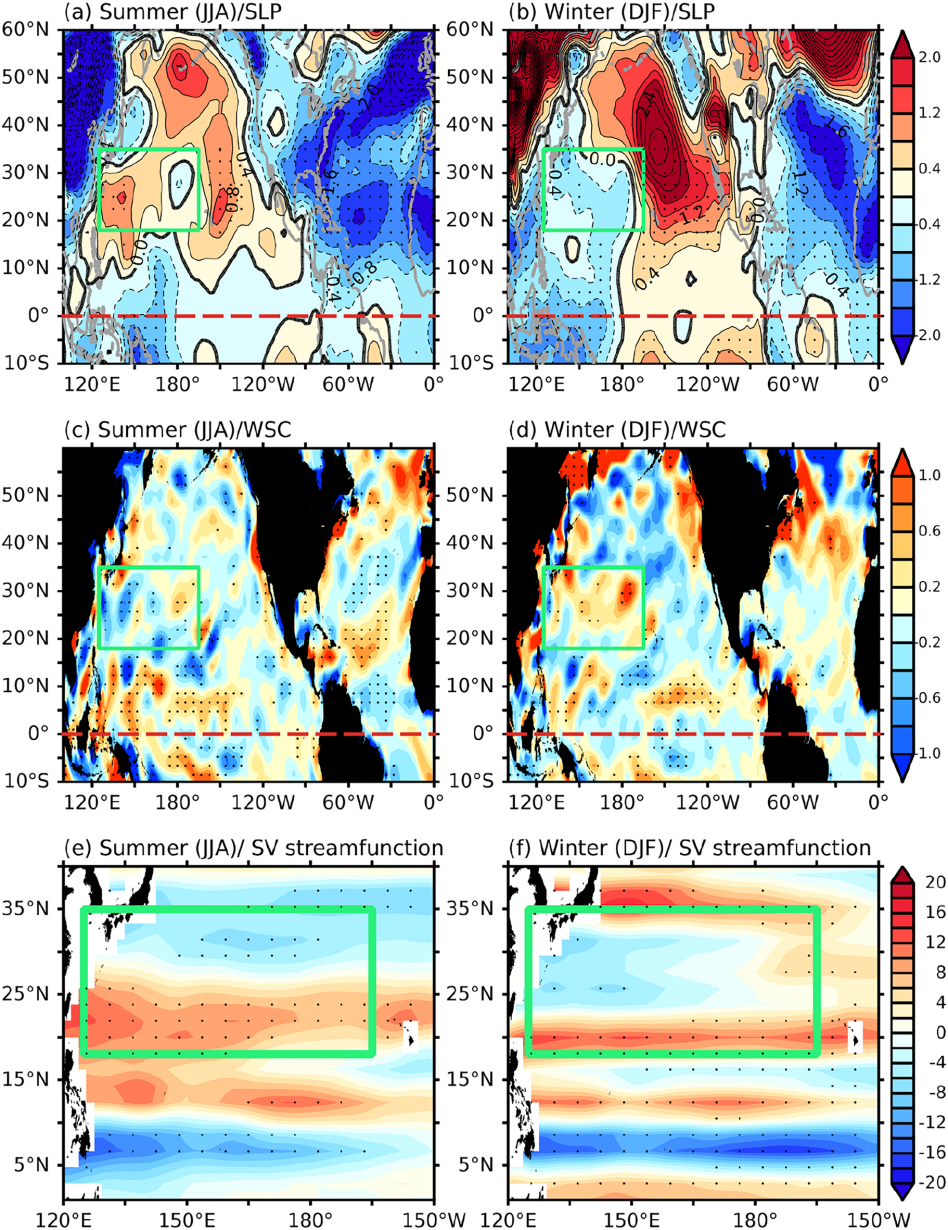
Regression of 7-year running-mean anomalies in SLP (contour, unit is hPa) onto the 7-year running-mean AMO index during summer (**a**) and winter (**b**) for the period 1948–2018. (**c,d**) Same as (**a,b**), but for wind stress curl (shading, unit is 10^–7^ N/m^3^). (**e,f**) Same as (**a,b**), but for Sverdrup streamfunction (unit is 10^6^ Sv). Red (blue) colors in (**e,f**) indicate clockwise (counter-clockwise) circulations. The red-dashed lines in (**a–d**) indicate the location of the equator, while the green boxes in all figures indicate the region bounded by 125° E–165° W and 18–35° N. The stippled areas in all figures indicate significance at the 90% confidence level determined using a Student’s t-test.

We next performed atmospheric analyses with the longer analysis period (i.e., 1948–2018) to understand the reasons why the AMO can induce different ocean current and SST responses in the WNP between winter and summer. To achieve this, we regressed the 7-year low-pass filtered sea level pressure (SLP) anomalies and WSCAs over the Atlantic–Pacific region onto the filtered AMO index during 1948–2018. We found that the atmosphere over the North Pacific responds differently to the AMO during summer and winter (Fig. 3). During summer, the AMO-regressed SLP anomalies reveal a strengthened subtropical high that covers almost the entire Pacific basin (Fig. 3a). Interestingly, negative SLP anomalies appear over the North Atlantic. This SLP regression pattern suggests that the positive AMO may induce anomalous ascent (and therefore negative SLP anomalies) over the North Atlantic and anomalous descent over the North Pacific (therefore positive SLP anomalies) though a mid-latitude trans-basin mechanism such as the one suggested by some previous studies^29,30^. This is confirmed in Fig. S4a where we regress the filtered omega velocity anomalies in a vertical cross-section averaged along the subtropical (15–30° N) Pacific–Atlantic sector onto the filtered AMO index. The regression is generally characterized by ascending motions over the North Atlantic and descending motions over the North Pacific. The anomalous descent intensifies the Pacific subtropical High during summer (Fig. 3a) and extends it further into the Western Pacific, giving rise to overall negative WSCA (color shadings in Fig. 3c) in the WNP. The overall negative WSCAs should intensify the NPSG and Kuroshio. Additionally, we present in Fig. 3e the regression of the filtered summer Sverdrup streamfunction over the North Pacific onto the filtered AMO index. A positive Sverdrup streamfunction in the subtropical region (between 15° and 30° N) indicates an increase in the strength of the Kuroshio (Fig. 1d,f).

During winter, the AMO-regressed SLP anomalies (Fig. 3b) are dominated by a poleward-propagating wavetrain pattern that emanates from the western tropical Pacific. This pattern suggests that the positive phase of the AMO may induce anomalous descent in the tropical Pacific during the winter season, in contrast to the descent in the North Pacific that occurred during the summer season. This is confirmed in Fig. S5b where we regress the filtered omega velocity anomalies over a vertical cross-section averaged along the tropical (0°–10° N) Pacific-Atlantic sector onto the filtered AMO index. The regression shows that anomalous descent occurred in the tropical central Pacific between 150° and 180° E, although the statistical significance area is limited to a small region. Since the winter atmosphere is barotropically more unstable (due to the strong vorticity gradient associated with the jet in winter and the strong upper-level divergent flow over the warm pool; not shown), the AMO-induced descent can excite a Rossby wavetrain propagating poleward into the North Pacific.

The AMO-regressed barotropic streamfunction and Z200 anomalies further reveal this Rossby wave pattern in the North Pacific during winter (Fig. S6b), which does not appear in summer (Fig. S6a). The wavetrain produces SLP anomaly centers over the NWP region (around 160° E and 25° N), the central North Pacific (around 150° W and 35° N), Gulf of Alaska (around 135° W and 50° N), and Northeastern Canada (around 55° W and 65° N). Such a wavetrain response in the North Pacific to the AMO has been documented in previous studies^34,35^. The anomalous low over the WNP, together with the slower mid-latitude westerlies caused by the anomalous high over the North Pacific, generate the positive WSCAs within the WNP region (Fig. 3d) associated with the seasonally reversed trends discussed earlier. The positive WSCAs should weaken the subtropical gyre circulation over the region, including the Kuroshio. Figure 3f confirms that, associated with the positive WSCAs over the 20° N–35° N region of the WNP, a weakened Sverdrup streamfunction appears. The negative Sverdrup streamfunction anomalies in the subtropical region indicate a weakened Kuroshio. When comparing the summer season (Fig. 3c), the most significant difference in the WSCA is observed in the 20° N–28° N region. Consequently, the greatest difference in the Sverdrup streamfunction also occurs in the same region. Therefore, it can be concluded that the seasonally reversed trends of the Kuroshio are specifically observed in the 20° N–28° N region due to these variability in the atmosphere. We also construct composites of the SLP, WSC, and Sverdrup streamfunction anomalies during the positive-AMO period (i.e., 1997–2018) for both summer and winter (see Fig. S7) to demonstrate that the AMO can trigger the seasonally reversed trends in the subtropical gyre. These figures exhibit a similar pattern to Fig. 3, showing a trans-basin impact pattern of the positive AMO over the North Pacific. Based on this result, it is evident that the AMO may indeed induce the seasonally reversed trends in the subtropical Northwestern Pacific. Affected by the length limitation of the observation data, the statistical significance area of the result is also limited. We further employed a coupled climate model to validate the plausibility of our theory.

**Figure 4:**
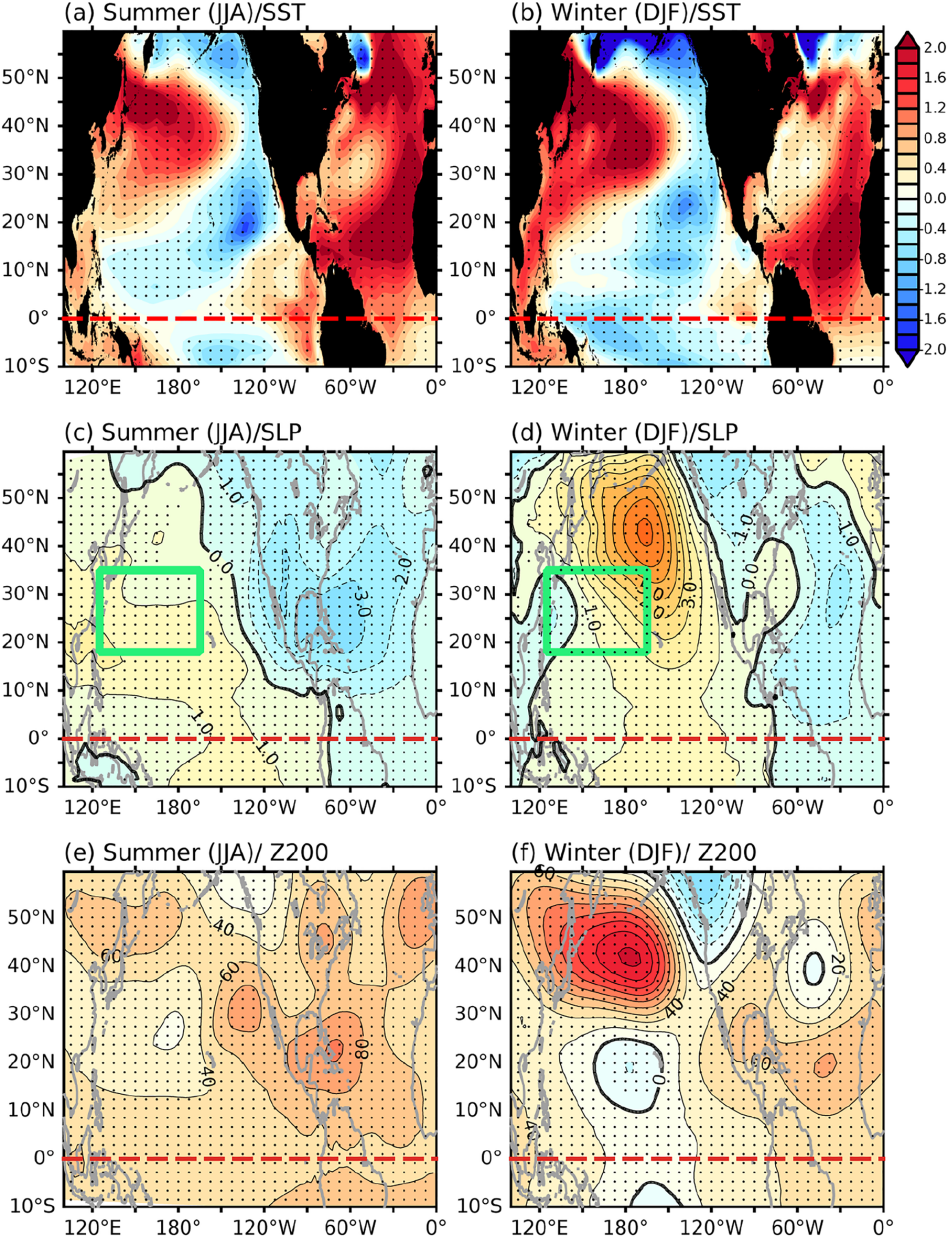
Ensemble mean SST (unit is °C) difference between positive and negative AMO experiments during summer (**a**) and winter (**b**). (**c–f**) Same as (**a,b**), but for SLP (unit is hPa) and Z200 (unit is m). The red-dashed lines in all figures indicate the location of the equator, while the green boxes in (**c,d**) indicate the region bounded by 125° E–165° W and 18–35° N. The stippled areas in all figures indicate significance at the 99% confidence level determined using a Student’s t-test.

We conducted pacemaker experiments using CESM1 to further investigate the trans-basin influences of the AMO on the Pacific climate. We nudged SSTAs in the North Atlantic (0–70° N) to simulate the positive AMO phase in one experiment (AMO-positive experiment) and the negative AMO phase in another experiment (AMO-negative experiment). Figure 4a,b displays the ensemble mean SST differences between the two experiments. In the North Atlantic, the SSTA differences resemble the observed AMO pattern, characterized by tropical and subpolar warming bands separated by a weaker-warming band in between. Figure 4c–f also shows the SLP and Z200 differences between the two experiments. During summer, the subtropical high across the entire Pacific basin strengthened, and negative SLP anomalies appeared over the North Atlantic (Fig. 4c). In winter, the SLP anomalies (Fig. 4d) were dominated by a poleward-propagating wavetrain pattern emanating from the western tropical Pacific (west of 150° E). The Z200 difference further revealed this Rossby wave pattern in the North Pacific during winter (Fig. 4f), but it was weaker during summer (Fig. 4e).

Recent studies have reported on the respective roles of the tropical and extra-tropical parts of the AMO’s SST anomaly pattern in affecting Pacific climate^30,34,36–38^. These studies suggested that the tropical Atlantic part of the AMO SST anomalies can first induce an anomalous descent over the tropical central Pacific, which the excite a poleward-propagating Rossby wave pattern into the North Pacific, similar to the PNA pattern^39^ (see Figs. 3b, 4d,f, Fig. S6b). However, the extra-tropical Atlantic part of the AMO SST anomalies can directly produce an atmospheric bridge from the North Atlantic to the North Pacific, with an anomalous ascent over the North Atlantic and an anomalous descent over the North Pacific (see Figs. 3a, 4c).

To further examine these seasonally varying forcing mechanisms, we examined the AMO-regressed omega vertical velocity anomalies along vertical cross-sections averaged over the subtropical (15–30° N) and tropical (0–10° N) Pacific-Atlantic Oceans during summer and winter from 1948 to 2018 (Figs. S4, S5). Our results reveal that the positive phase of the AMO induces similar anomalous descent in the tropical Pacific during both summer (Fig. S5a) and winter (Fig. S5b). However, the positive AMO forcing only lead to anomalous descent in the subtropical Pacific during summer (Fig. S4a), while no such descent is observed during winter (Fig. S4b). The main factor contributing to this discrepancy is the barotropic instability of the winter atmosphere, which enables the AMO-induced anomalous descent in the tropical Pacific to excite a poleward-propagating Rossby wave. This wave weakens the expected anomalous descent in the subtropical Pacific (around 150° E–150° W), which should have occurred due to the positive AMO forcing (see Fig. S4b). In contrast, during summer, the atmosphere exhibits greater barotropic stability, preventing the excitation of a similar propagating wave by the AMO-induced anomalous descent in the tropical Pacific. Consequently, the positive AMO-induced anomalous descent mainly occurs over the tropical Pacific during winter and over the subtropical Pacific during summer.

The seasonally reversed trend in SSTs off the East Asian coast can also be explained as an asymmetric Pacific response to the AMO modulated by the East Asian Monsoon. During summer, the anomalous surface high across the North Pacific in association with the positive AMO should intensify the summer southwesterly monsoon winds. We confirm in Fig. S8a that the East Asian summer monsoon winds exhibit a strengthening trend since the 1990s which can bring more moist air from the South China Sea region into the Taiwan Strait, reducing surface evaporation there. This explains how the summer warming trend is produced off the East Asian Coast (Fig. 1b). The suggested roles of surface heat fluxes are confirmed in Fig. S9, where we regress onto the AMO index the 7-year low-pass filtered anomalies of summer net heat flux (NHF) and its four components (i.e., latent heat flux, sensible heat flux, and shortwave and longwave radiation flux). The regressions show that the latent heat flux (Fig. S9g) and sensible heat flux (Fig. S9i) are the main contributor to the positive NHF forcing (Fig. S9a) of the summer SST warming trend off the East Asian coast. Apparently, the moister and warmer air imported by the anomalously strong summer monsoon winds dominates the effect of the stronger wind speeds which would be to cause a decrease in the surface evaporation and surface warming.

During winter, the AMO-induced wavetrain produces anomalously low surface pressures to the east of Taiwan, resulting in anomalous northerlies on its western flank, which strengthens the winter monsoon. We also confirmed that the East Asian winter monsoon has a strengthening trend since 1990s (Fig. S8b). The regression analysis with the winter surface heat flux components (Fig. S9 right panels) further indicates that the strengthened winter monsoon increases surface evaporation, primarily through the latent heat flux component, resulting in winter cooling of SSTs observed off the East Asian coast. Unlike the strengthened summer monsoon, the strengthened winter monsoon affects the surface evaporation through changes in wind speed rather than changes in the humidity. Although Fig. S9 is almost not statistically significant around the Taiwan Strait, these findings suggest a potential influence of the AMO around the Taiwan Strait.

In addition to the surface heat flux, the East Asian monsoon also plays a role in modulating the strength of the China Coastal Current (CCC), which could contributes to the cooling trend in winter^40^. The CCC is a narrow band of southward currents that appears only during winter off the China coast (Fig. S10). Driven by the East Asian winter monsoon, the CCC can transport cool water from the Yellow Sea and East China Sea into the South China Sea through the Taiwan Strait^41,42^. We found that the winter CCC speed averaged over the TS transect line (black line in Fig. S10) shows a strengthening trend (of − 0.01 cm/s/year) during the 1993–2018 (Fig. S8c). The CCC current speed and East Asia winter monsoon index are significantly correlated (R = 0.52 with P < 0.01) in the TS transect line. A stronger East Asian winter monsoon causes a stronger CCC, which resulted in more cold water flowing southward into the Taiwan Strait, contributing to the observed winter cooling trend there. The CCC can serves as a conduit for transmitting the monsoon-induced changes more prominently to SST, thereby influencing the alteration of SST. As the CCC can affects SST in the Taiwan Strait, the AMO’s influence on Western Pacific SST trends becomes particularly pronounced in this region.

## Discussion and summary

**Figure 5:**
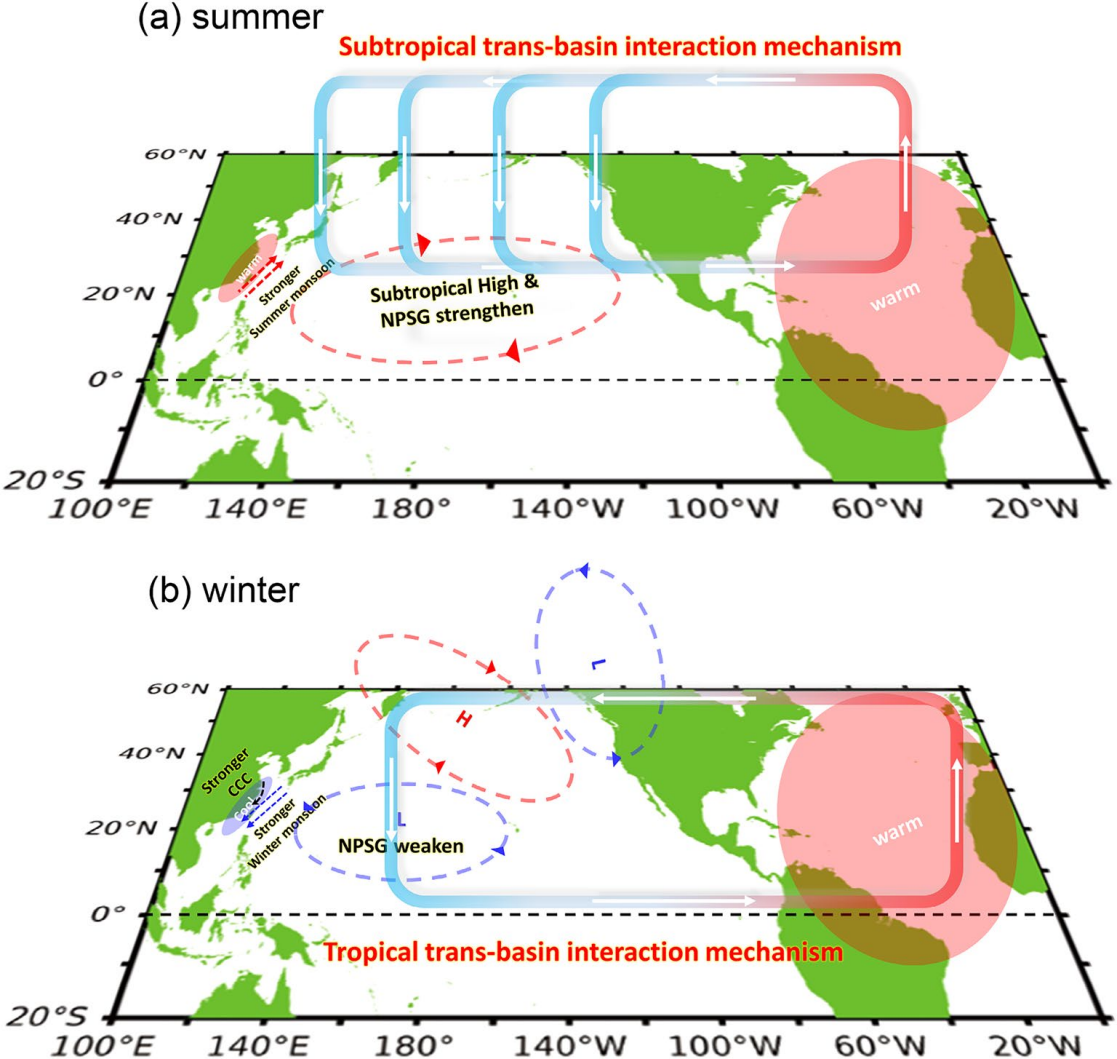
Diagram illustrating the processes enable the asymmetric Atlantic–Pacific trans-basin interactions during boreal summer (**a**) and winter (**b**). During summer (**a**), the positive AMO induces anomalous ascent over the North Atlantic and anomalous descent over the subtropical North Pacific. This subtropical trans-basin interaction mechanism directly intensifies the Pacific Subtropical High, which then produces a strengthening trend in the gyre circulation in the WNP through changes in wind stress curls and a warming trend in SST off the East Asian coast via the strengthening of the East Asian summer monsoon. During winter (**b**), the positive AMO first induces anomalous descent over the tropical North Pacific. This tropical trans-basin interaction mechanism subsequently excites a wavetrain that propagates into the North Pacific. The wave train consists of an anomalous cyclone north of 20° N in the WNP, which causes a gyre weakening trend through wind stress curl anomalies and a SST cooling trend off the Asia coast via a strengthening of the East Asian winter monsoon and associated China Coastal Current.

Using statistical analyses, we find seasonally reversed trends in surface currents and SSTs in the WNP from 22° to 28° N since the 1990s. However, given the constrained length of the observational data, we extend our analysis by employing CESM1 pacemaker experiments. These experiments serve to validate that the observed trends stem from the North Pacific’s asymmetric responses to the AMO during both the summer and winter seasons. Figure 5 summarizes and illustrates the series of processes involved in the asymmetric trans-basin interactions. As the AMO changed to a positive phase beginning in the middle 1990s, the warmer SSTs in the North Atlantic induced an anomalous descent over the North Pacific during the summer season. This subtropical trans-basin interaction mechanism directly strengthens the Pacific subtropical High, which then produces a strengthening of the gyre in the WNP through changes in wind stress curls and a warming trend in ocean surface temperatures off the East Asian coast (Fig. 5a) through enhancement of East Asian summer monsoon. During winter (Fig. 5b), the positive phase of the AMO induces an anomalous descent over the Tropical Pacific that subsequently excites a poleward-propagating Rossby wavetrain into the North Pacific. The wave train produced by this tropical trans-basin interaction mechanism consists of an anomalous surface cyclone north of 20° N in the WNP. This anomalous cyclone produces a gyre weakening trend through wind stress curl changes and a cooling trend off the Asian coast through enhancement of the East Asian winter monsoon and the associated CCC. The seasonally reversed trends observed in the WNP are thus a result of asymmetric Atlantic-Pacific trans-basin interactions between summer and winter.

Previous studies have pointed out that global warming can enhance inter-basin interaction between the pantropical (including Pacific) and Northern Atlantic regions, especially after the 1990s^29,43,44^. Therefore, it is likely that climate change in the Atlantic will become increasingly important for the Pacific climate.

## Data and methods

### Data sets

To study ocean current and SST variations in the WNP region, we use high-resolution monthly reanalysis data provided by the Japan Coastal Ocean Predictability Experiment Reanalysis (JCOPE2). This ocean data set has a domain that covers the WNP from 10.5° to 62° N and from 108° to 180° E with a resolution of 1/12° in the horizontal and 46 sigma levels in the vertical^45^. We verify the results related to ocean surface currents with the observations provided by the Archiving, Validation and Interpretation of Satellite Oceanographic Data (AVISO) and SSTs with the optimum interpolation SST (OISST) from the NOAA National Centers for Environmental Information. These two observational datasets have a global horizontal resolution of 0.25°$\times$ 0.25°. For the analyses related to the trans-basin interactions between Pacific and Atlantic Oceans, atmospheric information over these two basins are provided by National Centers for Environmental Prediction/National Center for Atmospheric Research (NCEP/NCAR) reanalysis 1. The NCEP/NCAR reanalysis 1 has a 1.875° $\times$ 1.875° horizontal resolution. In this study, the analysis periods are: 1993–2018 for the regional analyses using the JCOPE2, AVISO, OISST and 1948–2018 for the trans-basin analyses using NCEP/NCAR reanalysis I. Anomalies are defined as the deviations from the monthly climatology calculated using the respective analysis periods (i.e., 1993–2018 or 1948–2018). In addition, the linear trends during 1948–2018 were removed from NCEP/NCAR reanalysis I data (see Figs. 2, 3, Figs. S2, S4–S9).

### Climate indices

Four climate indices were used in the study: the AMO, PDO, East Asia summer monsoon index, and East Asia winter monsoon index. The AMO index^46^ is obtained from NOAA’s Physical Sciences Division and is calculated as the de-trended SST anomalies averaged over the North Atlantic from the equator to 70° N. The PDO index is obtained from the Joint Institute for the Study of the Atmosphere and Ocean (JISAO) and is the principal component of the first leading Empirical Orthogonal Function mode of the North Pacific SST anomalies^47^. The East Asia summer and winter monsoon indices^48–51^ are used to quantify the strength of the monsoon during the two seasons. The East Asia summer monsoon index is defined as surface meridional wind averaged over the region between 10–40° N and 110–140° E, whereas the East Asia winter monsoon index is defined as surface meridional wind averaged over the region between 20–40° N and 100–140° E.

### The CEMS1 pacemaker experiments

All the experiments in this study were conducted using the CEMS1.2.2 (for more details, see https://www.cesm.ucar.edu/models/cesm1.2/). The experiments utilized the B1850C5CN (fully coupled) compset of the CESM1.2.2, with the f19_g16 model configuration. This configuration has a horizontal resolution of approximately 2° and a vertical resolution of 30 vertical levels in the Community Atmospheric Model version 5.3 (CAM5.3), and a horizontal resolution of approximately 1° and 60° vertical levels for the Parallel Ocean Program version 2 (POP2).

The experiments consisted of an AMO-positive and an AMO-negative experiment. In the pacemaker experiments, the North Atlantic (0–70° N) SST was restored to the model climatology plus the observed AMO-positive or AMO-negative SST pattern. The AMO’ SSTA pattern was obtained by regressing North Atlantic SSTAs onto the AMO index using 1900–2018 observations. The restoring timescale was set as 10 days, and 5 ensemble members were generated for each AMO-positive and AMO-negative experiment in this study.

The climatological mean states and the external forcings (e.g., greenhouse gas, solar forcing, and aerosol) were fixed at preindustrial levels. Each member of the experiments was integrated for 110 years, with the model output from the last 80 years analyzed.

### Significance test

The statistical significance of correlation is examined using the two-tailed P-values from a Student’s t-test, and the degrees of freedom used in the test is determined by considering the auto-correlation of the tested variables. The calculation of the effective degrees of freedom (*df*) is determined according to the method proposed by Bretherton et al.^52^ and can be expressed as:

$$df = {\text{ N}} \times \left( {\left( {{1} - {\text{r1r2}}} \right)/\left( {{1} + {\text{r1r2}}} \right)} \right) - {2}.$$

The length of the year analyzed is denoted by N, while r1 and r2 represent the lag one autocorrelation coefficients of the two series under consideration in the regression or correlation analysis.

### Supplementary Information

Supplementary Figures.

## Data Availability

The JCOPE2 Reanalysis data was obtained from https://www.jamstec.go.jp/jcope/htdocs/e/distribution/index.html. The AVISO, OISST, and NCEP r1 data were downloaded from https://www.aviso.altimetry.fr/en/data.html, and https://data.nodc.noaa.gov, respectively. The AMO and PDO indices were obtained from http://www.esrl.noaa.gov/psd/data/timeseries/AMO/, and http://research.jisao.washington.edu/pdo/, respectively. The CESM1 pacemaker simulations data used in this study can be obtained by contacting Yong-Fu Lin at yongfulin0711@gmail.com.
