# Supplementary material for: Seasonally-reversed trends in the subtropical Northwestern Pacific linked to asymmetric AMO influences
Source: Sci Rep. 2023 Aug 23;13:13735. doi: 10.1038/s41598-023-40979-9 (PMC10447491; doi:10.1038/s41598-023-40979-9)
Supplement: Supplementary file 1 — Supplementary Figures. [file 41598_2023_40979_MOESM1_ESM.docx]

**Supporting Information for**

**Seasonally-reversed trends in the subtropical Northwestern Pacific linked to asymmetric AMO influences**

Yong-Fu Lin^1^, Chuen-Teyr Terng^2^, Chau-Ron Wu^3,4^, and Jin-Yi Yu^1*^

^1^Department of Earth System Science, University of California, Irvine, CA, USA

^2^Central Weather Bureau, Taipei, Taiwan

^3^Department of Earth Sciences, National Taiwan Normal University, Taipei, Taiwan

^4^Research Center for Environmental Changes, Academia Sinica, Taipei, Taiwan

*Correspondence to jyyu@uci.edu

**Contents of this file**

Figures S1-S10


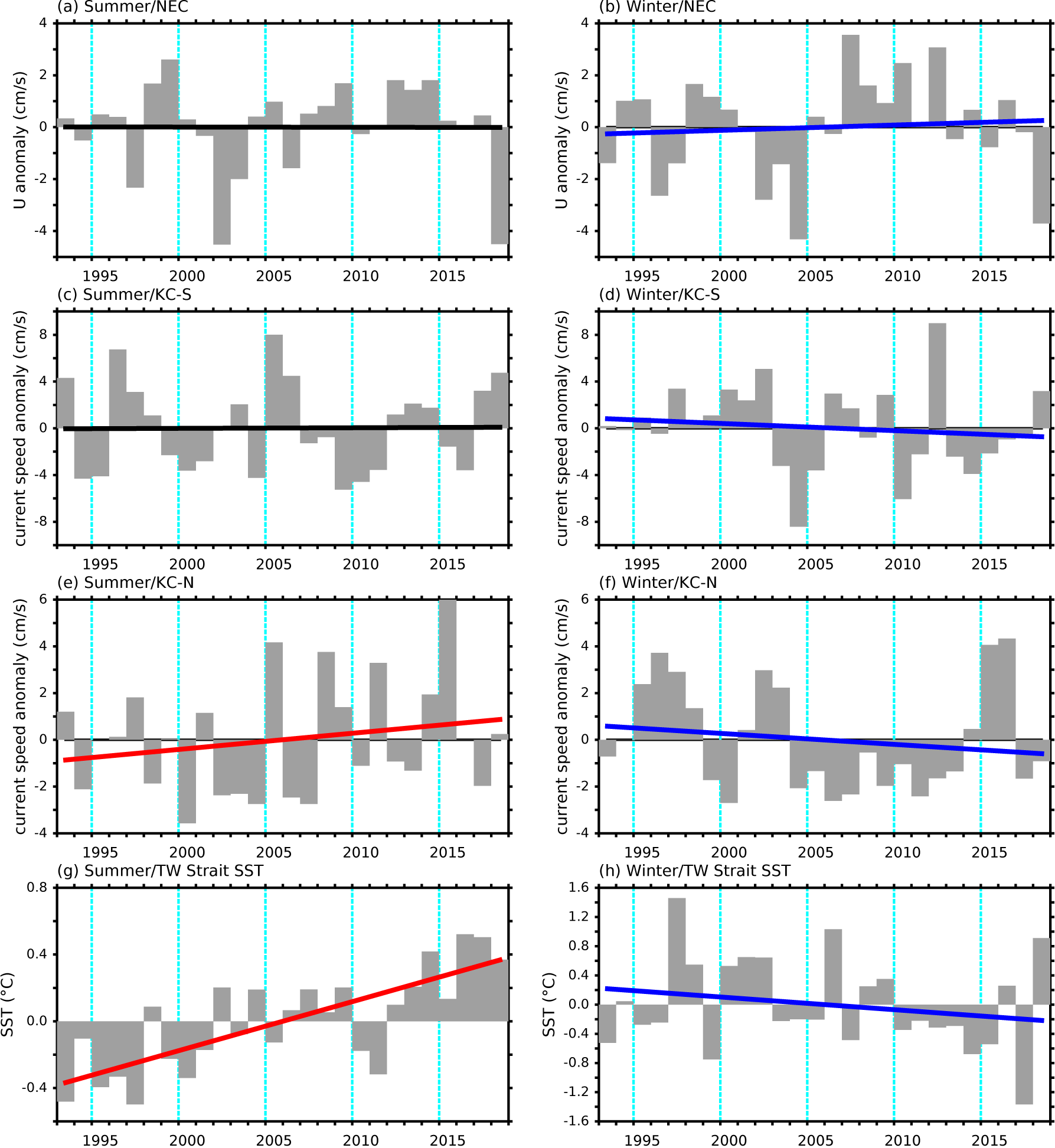


Figure S1. Time series of the zonal current speed averaged over the upper-ocean (0-200m) in the NEC region (green box in Figure 1a) are shown for summer (a) and winter (b). (c)-(d) and (e)-(f) show the same analysis for the Kuroshio Current, with average speeds computed upstream (KC-S line in Figure 1a) and downstream (KC-N line in Figure 1a), respectively. Time series of sea surface temperature (SST) averaged over the sections of Taiwan Strait (TS lines in Fig. 1a or S10) are also shown for summer (g) and winter (h). The red and blue lines indicate increasing (or enhancing) and decreasing (or weakening) trends, respectively.


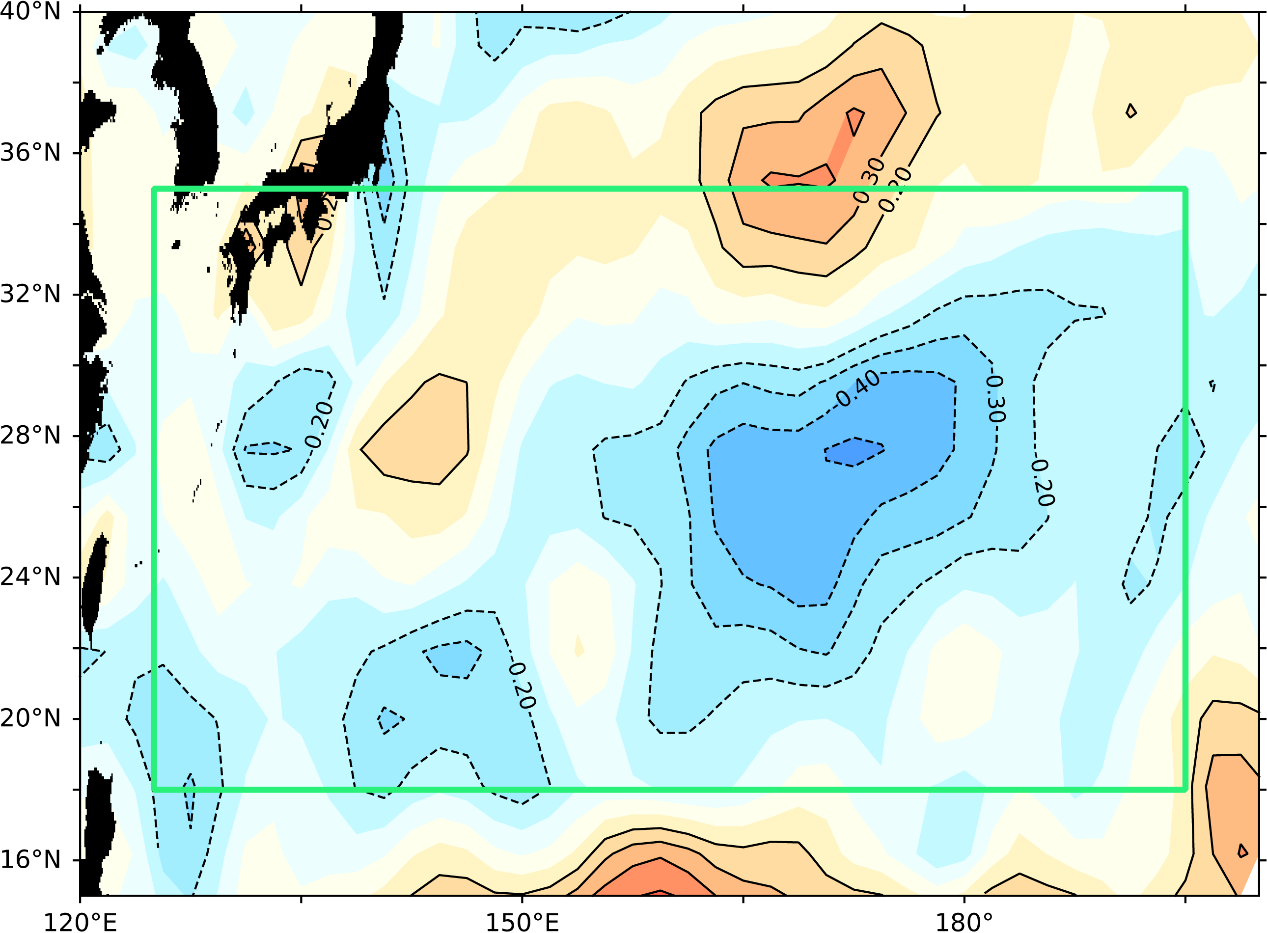


Figure S2. Correlation coefficients between the monthly upper-ocean (0-200m) Kuroshio Current speed averaged along the KC-N line (see Figure 1a) and wind stress curl (WSC) over the Western North Pacific during 1993-2018. Contours indicate the 90% confidence level determined using a Student’s t-test. Green box indicates the region (125°E–165°W and 18–35°N) selected for further WSC analyses.


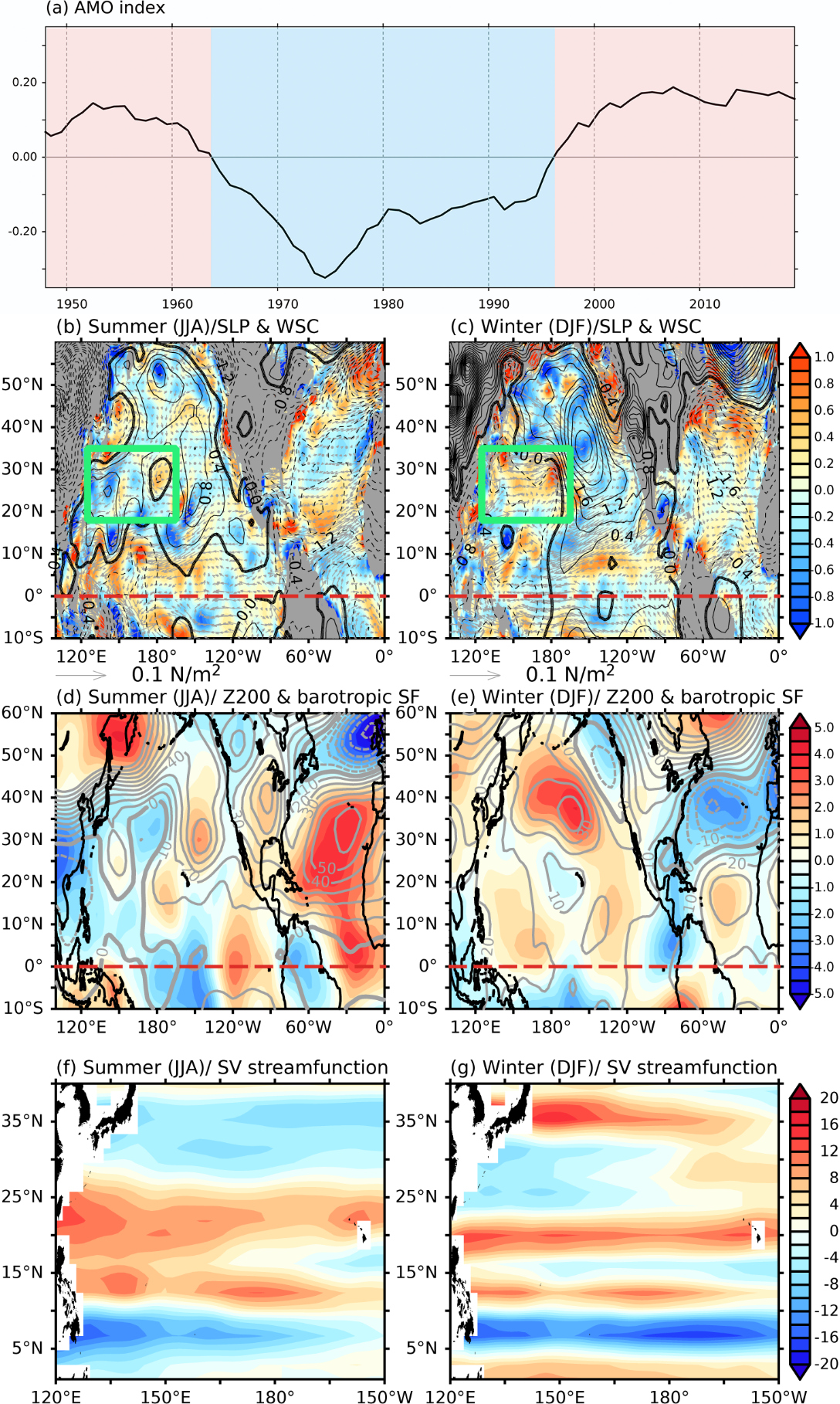


Figure S3. Time series of the yearly AMO index values (a seven-year running mean has been applied). The AMO positive (negative) periods are marked by red (blue) shadings.


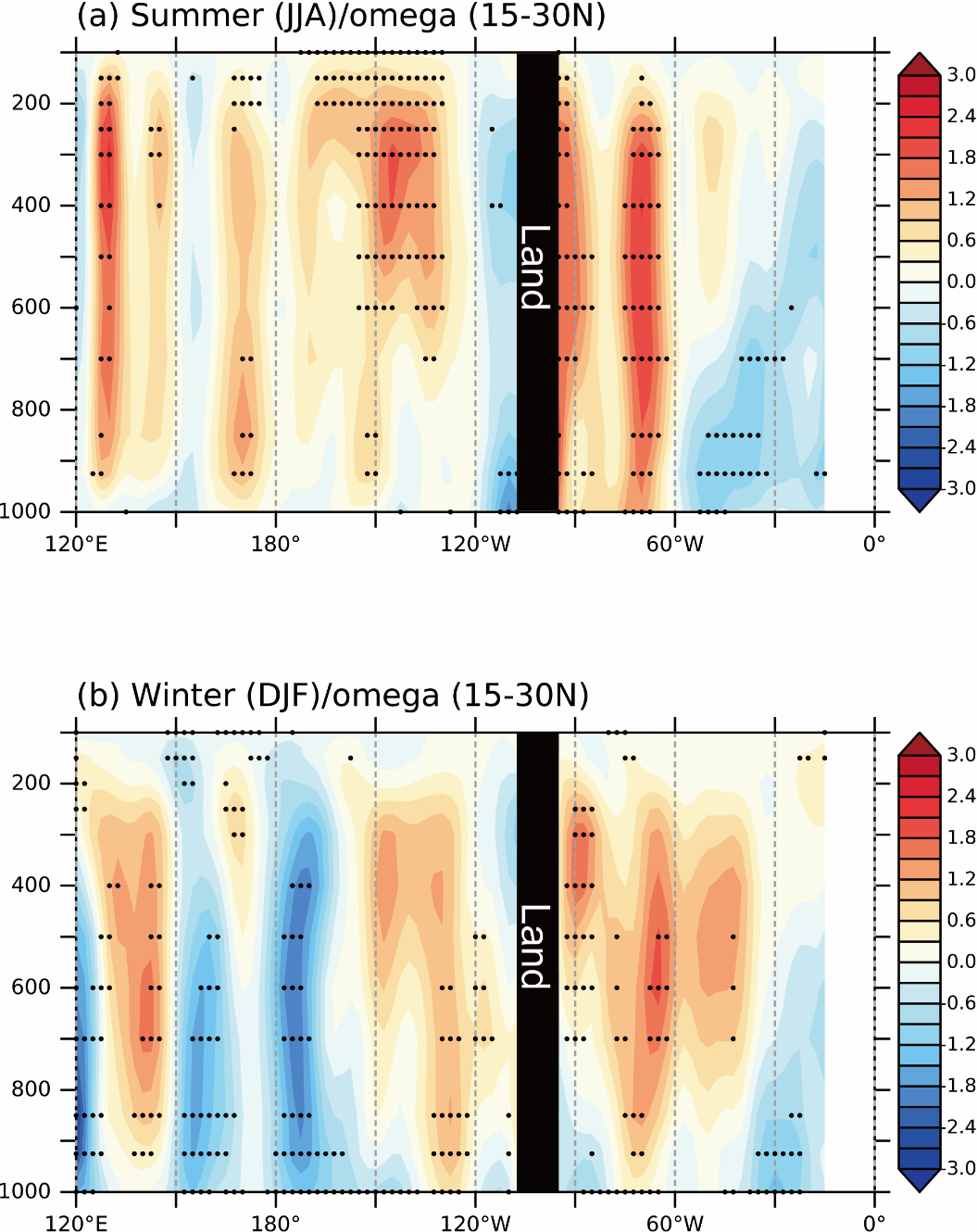


Figure S4. The AMO-regressed omega vertical velocity (10^-2^ Pa/s) anomalies over the a vertical cross-section averaged along the subtropical (15-30°N) Pacific-Atlantic Oceans in summer (a) and winter (b) during 1948-2018. A 7-yr running mean is applied to both the AMO index and the omega velocity before the regressions are performed. Black areas indicate the American continent. The stippled areas in all figures indicate significance at the 90% confidence level determined using a Student’s t-test.


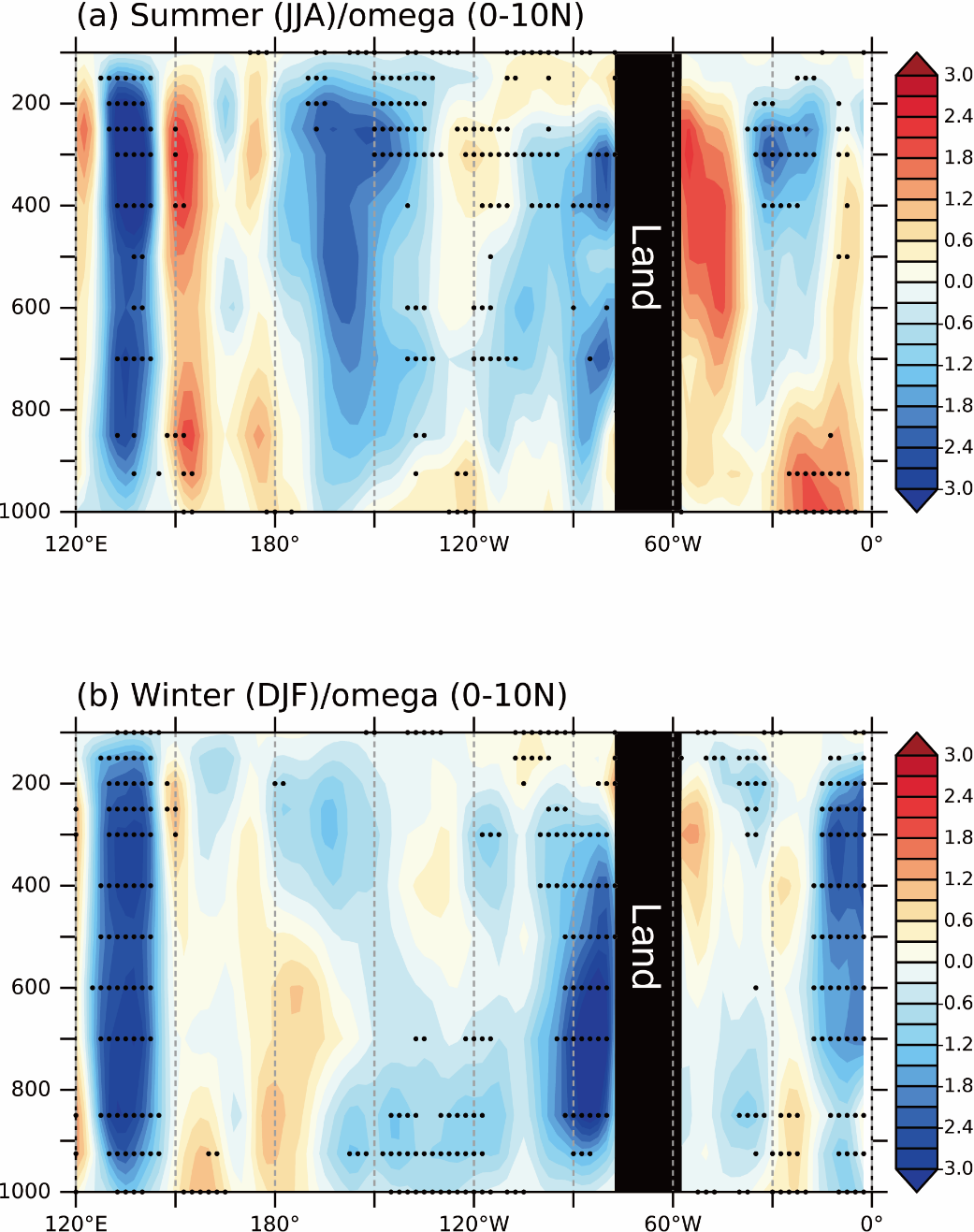


Figure S5. The AMO-regressed omega vertical velocity (10^-2^ Pa/s) anomalies in a vertical cross-section averaged along the tropical (0-10°N) Pacific and Atlantic Oceans in summer (a) and winter (b) during 1948-2018. A 7-yr running mean is applied to both the AMO index and the omega velocity before the regressions are performed. Black areas indicate the American continent. The stippled areas in all figures indicate significance at the 90% confidence level determined using a Student’s t-test.


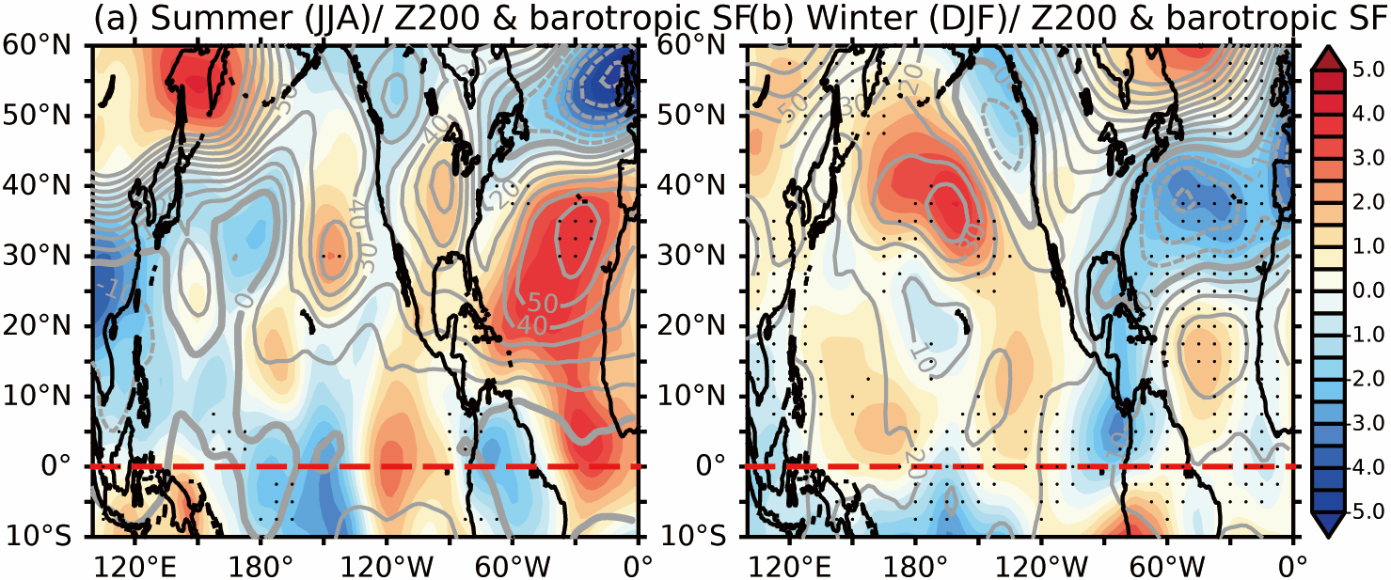


Figure S6. Regression of 7-year running mean Z200 (contour, unit in m) and barotropic streamfunction (colors, unit in 10^6^ m^2^/s) onto 7-year running mean AMO index in summer (a) and winter (b) during 1948-2018. Red dash lines indicate the location of the equator. The stippled areas in all figures indicate significance at the 90% confidence level determined using a Student’s t-test.


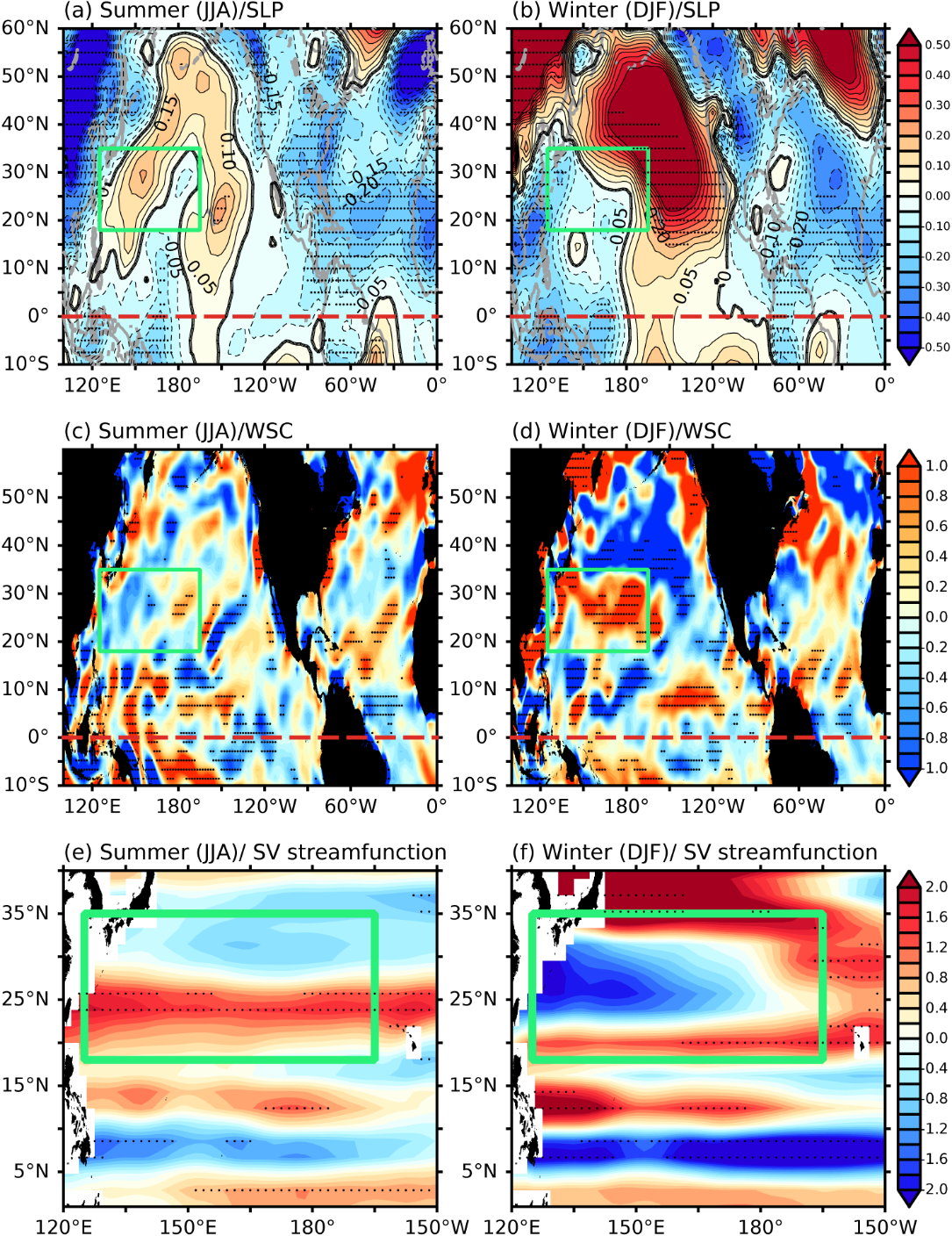


Figure S7. Composite of anomalies in SLP (contour, unit is hPa) during summer (a) and winter (b) for the period 1997-2018. (c)-(d) same as (a)-(b), but for wind stress curl (shading, unit is 10^-7^ N/m^3^). (e)-(f) same as (a)-(b), but for Sverdrup streamfunction (unit is 10^6^ Sv). Red (blue) colors in (e)-(f) indicate clockwise (counter-clockwise) circulations. The red-dashed lines in (a) - (d) indicate the location of the equator, while the green boxes in all figures indicate the region bounded by 125°E–165°W and 18–35°N. The stippled areas in all figures indicate significance at the 90% confidence level determined using a Student’s t-test.


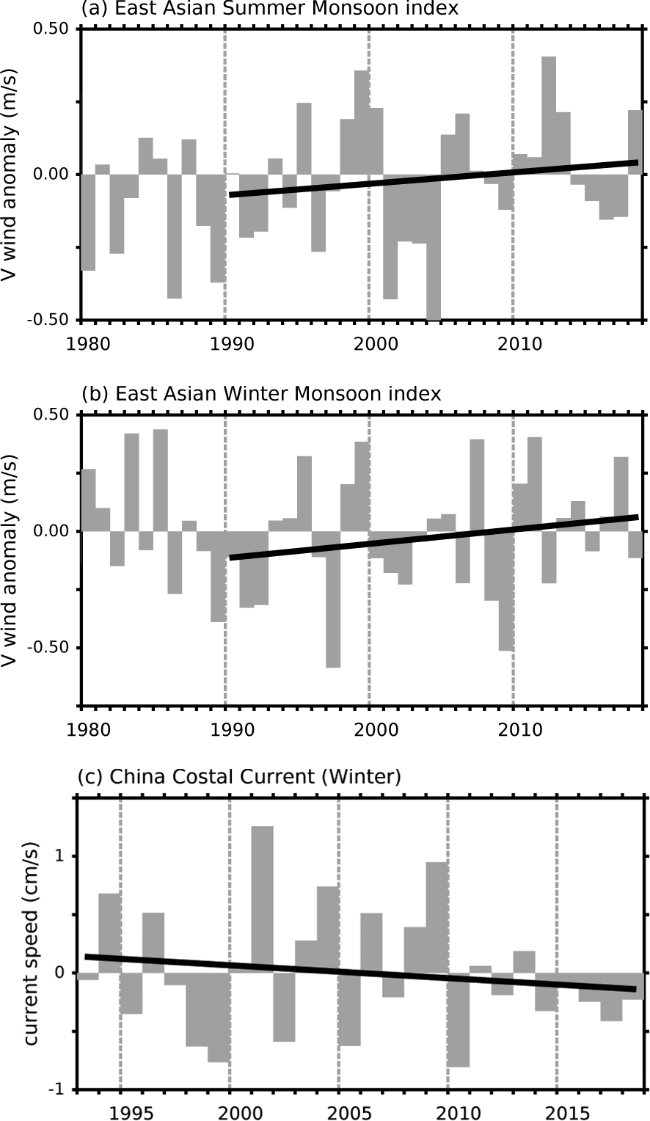


Figure S8. Time series (bars) and linear trends (black lines) of the yearly (a) East Asian summer monsoon index, (b) winter monsoon index, and (c) winter Coastal China Current speed averaged across the TS line (black line in Figure S10).


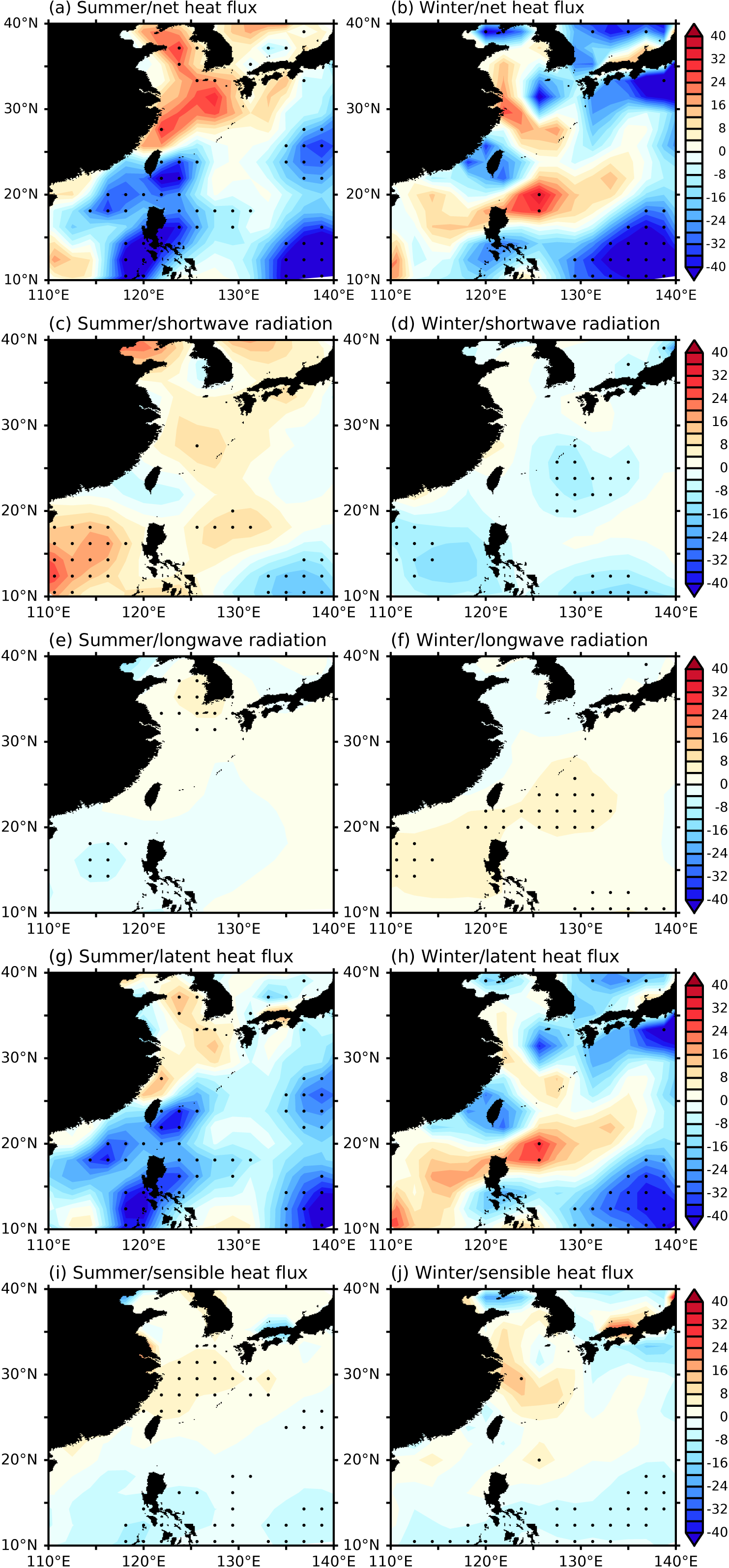


Figure S9. Regression of the 7-year running mean net heat flux (unit is W/m^2^) onto the 7-year running mean AMO index during summer (a) and winter (b) for the period 1948-2018. (c)-(d), (e)-(f), (g)-(h), and (i)-(j) same as (a)-(b), but for shortwave radiation, longwave radiation, latent heat flux, and sensible heat flux (all units are W/m^2^), respectively. Positive values indicate fluxes from the atmosphere to the ocean and vice versa for negative values. The stippled areas in all figures indicate significance at the 90% confidence level determined using a Student’s t-test.


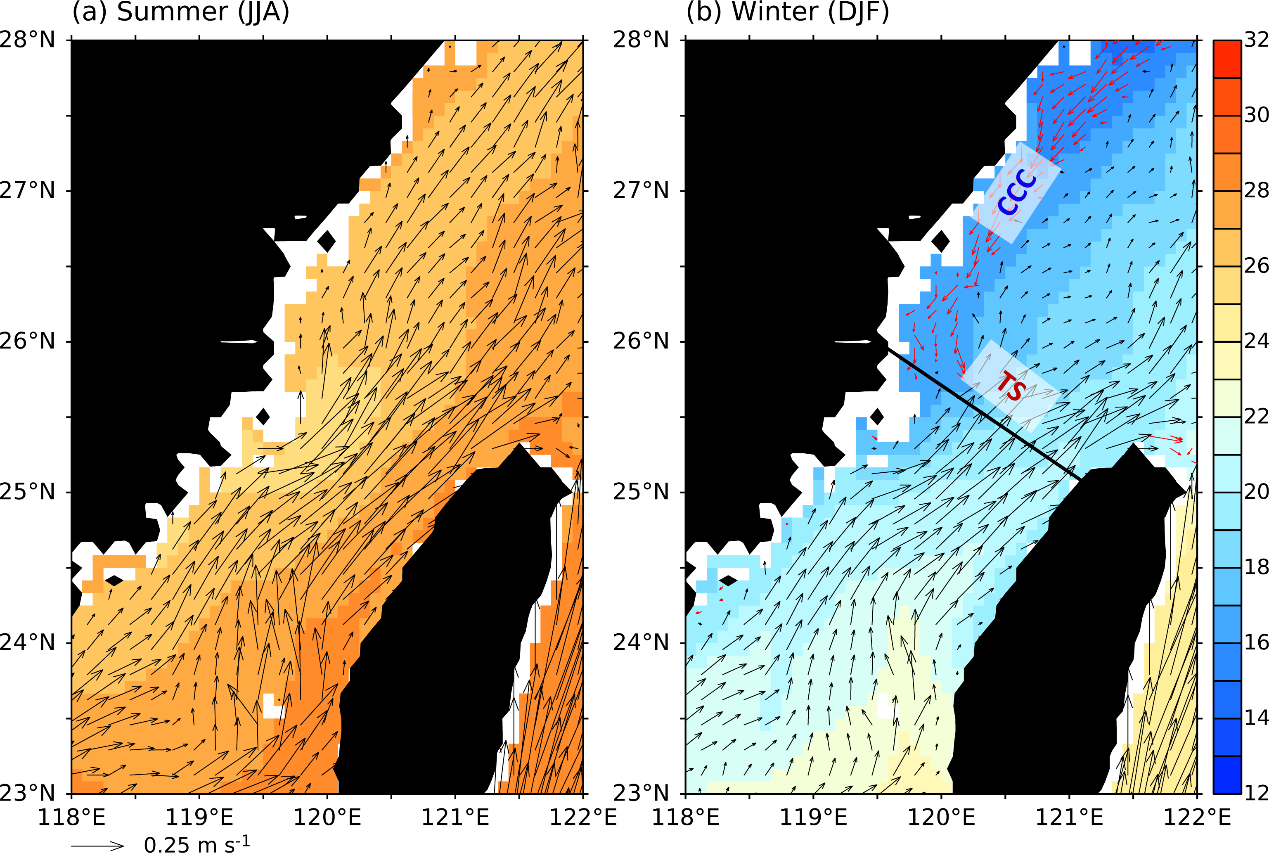


Figure S10. Seasonal mean SST (colors) and upper-ocean (0-200m) current speed (vectors) during summer (a) and winter (b) from JCOPE2 reanalysis data for the period 1993-2018. The red vectors in (b) indicate the China Coastal Current (CCC). The “TS” line marks the Taiwan Strait transect used in the analyses.
